# Use of direct oral anticoagulants does not significantly increase delayed bleeding after endoscopic submucosal dissection for early gastric neoplasms

**DOI:** 10.1038/s41598-021-88656-z

**Published:** 2021-04-30

**Authors:** Jinju Choi, Soo-Jeong Cho, Sang-Hoon Na, Ayoung Lee, Jue Lie Kim, Hyunsoo Chung, Sang Gyun Kim

**Affiliations:** 1 Division of Gastroenterology, Department of Internal Medicine and Liver Research Institute, Seoul National University Hospital, 101, Daehak-ro Jongno-gu, Seoul, 03080 Korea; 2 Division of Cardiology, Department of Internal Medicine, Seoul National University Hospital, Seoul, Korea

**Keywords:** Oesophagogastroscopy, Risk factors, Gastrointestinal diseases, Adverse effects, Therapeutic endoscopy

## Abstract

Direct oral anticoagulants (DOACs) are widely prescribed for the prevention of stroke in elderly patients with atrial fibrillation and approved indication for DOAC has been expanded. We aimed to evaluate the risk of delayed bleeding in patients who had taken DOAC and underwent endoscopic submucosal dissection (ESD) for gastric neoplasms. We included consecutive patients who underwent ESD between January 2016 and July 2019 in Seoul National University Hospital. Patients were divided into four groups (no med; no medication, DOAC, WFR; warfarin, anti-PLT; anti-platelet agent) according to the medications they had been taken before the procedure. We defined delayed bleeding as obvious post-procedural gastrointestinal bleeding sign including hematemesis or melena combined with hemoglobin drop ≥ 2 g/dL. Among 1634 patients enrolled in this study, 23 (1.4%) patients had taken DOAC and they usually stopped the medication for 2 days before the ESD and resumed within 1 or 2 days. We compared rates of delayed bleeding between groups. Delayed bleeding rates of the groups of no med, DOAC, WFR, and anti-PLT were 2.1% (32/1499) 8.7% (2/23), 14.3% (2/14), 11.2% (11/98), respectively (*P* < 0.001). However, there was no difference of delayed bleeding rate between no med and DOAC group after propensity score matching (no med vs DOAC, 1.7% vs 10.0%, *P* = 0.160). Taking DOAC was not associated statistically with post-ESD bleeding when adjusted by age, sex, comorbidities and characteristics of target lesion (Adjusted Odds Ratio: 2.4, 95% Confidence intervals: 0.41–13.73, *P* = 0.335). Crude rate of bleeding in DOAC users seemed to be higher than no medication group after performing ESD with 2 days of medication cessation. When adjusted by age, sex, and comorbidity, however, this difference seems to be small, which suggests that gastric post-ESD bleeding may be influenced by patients’ underlying condition in addition to medication use.

## Introduction

Endoscopic submucosal dissection (ESD) is a widely accepted curative treatment for early gastric neoplasms, including gastric adenomas and early gastric cancers (EGCs) that have a negligible risk of lymph node metastasis. ESD is an effective technique that enables the complete resection of even large gastric neoplasms; however sufficient experience and knowledge are required to avoid such complications as bleeding and perforation^1,2^. Bleeding is the most frequent major complication of ESD. During ESD, hemostasis is performed for active bleeding or visible vessels, and almost all occurrences of bleeding can be effectively controlled^3^. Intravenous administration of a proton pump inhibitor on the day of ESD followed by an oral proton pump inhibitor for 4–8 weeks post-ESD, are used to treat post-ESD ulcers and as prophylaxis for delayed bleeding^1^. Furthermore, the management of anti-thrombotic agents prior to elective ESD is important to prevent procedure-related bleeding.

The use of direct oral anticoagulants (DOACs) has been recently increasing to prevent thromboembolism in patients with non-valvular atrial fibrillation (Afib). In Korea, DOACs have been widely used after the Korean National Health Insurance Service approved the listing of DOACs in its formulary in 2015. DOACs increase the risk of GI bleeding, while they were reported to be non-inferior to WFR in preventing thromboembolic complications^4–6^. Therefore, the risk of DOAC use for post-ESD bleeding must be evaluated. Although a guide for the peri-procedural management of DOACs is necessary, there is no current consensus on their use in such scenarios. A multidisciplinary approach for peri-procedural anticoagulation management is recommended to address the risk of bleeding and thrombosis, as well as to consider the half-life of the drug and the patient’s creatinine clearance^7–10^. When to stop and re-start a DOAC differs according to its composition. In general, for patients with normal renal function, a DOAC is stopped 1–3 days prior to ESD, but there is little guidance about when to stop and resume a DOAC before and after ESD, respectively^8,11,12^.

We aimed to evaluate the risk of delayed bleeding after ESD in patients who had taken a DOAC and the associated factors that influence the risk of bleeding. We ultimately discuss the periprocedural management of DOACs in patients who are scheduled to undergo ESD for gastric neoplasms.

## Methods

### Study population and data

This retrospective study was conducted by a manual review of the electronic medical record from Seoul National University Hospital (SNUH). Patients who were diagnosed with EGC or gastric adenoma (low or high grade), and subjected to ESD were enrolled between January 2016 and July 10th 2019. ESD was performed for well- or moderately-differentiated tubular adenocarcinoma no larger than 3 cm, which is generally confined to the mucosa^13,14^. All gastric adenomas regardless of size and morphology are considered candidates for ESD^15,16^. Patients lacking a medication history, medical history, or pathology report were excluded. Cases of recurred EGC or adenoma at a previous ESD site were excluded. Patients were divided into four groups [no medication (no med), DOAC, WFR, and anti-platelet (anti-PLT) agent], according to the periprocedural medication administered. Basic demographic data, comorbidities, medications, laboratory data, endoscopic findings, histopathology of ESD specimen, delayed bleeding events, red blood cell (RBC) transfusions, second-look endoscopy and occurrence of endoscopic hemostasis were reviewed retrospectively. Delayed bleeding was defined as a sign of upper gastrointestinal bleeding including hematemesis or melena with a drop in hemoglobin greater than or equal to 2 g/dL and/or a demand for RBC transfusion or emergent endoscopy within 4 weeks of the completion of the procedure^17^. The Institutional Review Board (IRB) of SNUH approved the study protocol (IRB number: H-1905-158-1035). This study was conducted according to the principles of the Declaration of Helsinki. Informed consent was obtained from all study participants.

### Periprocedural use of anti-PLT agents or anticoagulants

As ESD for gastric neoplasms is elective procedure, ESD was performed after the cessation of anticoagulation and there was no continuous user of anticoagulant. Patients who took dual anti-thrombotics quit all drugs before the procedure after a discussion with a cardiologist. The timing of cessation and resumption of the drugs was determined after cardiology consultation but it could be varied according to the patient’s compliance. In clinical practice of cardiology in SNUH, heparin bridging therapy was not a routine process in patients who took anticoagulation for atrial fibrillation. Medication use and duration of use (including cessation of and resumed use), medical ingredients, and dosages were reviewed. Medical records, electronic orders (by a physician), and preadmission medical information were reviewed to compile detailed information about each patient’s medication history. DOAC users were defined as patients who had been administered dabigatran, rivaroxaban, edoxaban, or apixaban before and after ESD. Anti-PLT users were defined as patients who had been given one or two anti-PLT agents prior to ESD and the duration of periprocedural cessation was no longer than 3 days prior to ESD. This group included continuous anti-PLT users. Screened anti-PLT agents included aspirin, clopidogrel, cilostazol, ticlopidine, prasugrel, and ticagrelor. WFR users were patients who had been given WFR before and after ESD.

### Statistical analysis

To evaluate the influence of DOACs on delayed bleeding after ESD, the rate of delayed bleeding was compared among the groups using Fisher’s exact test. Continuous variables, reported as means or median with standard deviations or ranges were analyzed using ANOVA or Kruskal–Wallis test. Analysis of matched cohorts was designed to compare the rates of delayed bleeding between DOAC users and those not taking medication (no med group) using 1:3 propensity score matching without replacement and with a caliper of 0.2. Matching variables included demographic characteristics (age, sex), body mass index (BMI), HAS-BLED (hypertension, abnormal renal or liver function, stroke, bleeding history or predisposition, labile international normalized ratio, elderly, drugs or alcohol concomitantly), clinicopathologic features of the target lesion (location, size of the specimen, invasion depth, ulcer presence and histology). Evaluation of factors associated with delayed bleeding after ESD was assessed using multivariable logistic regression. Predictors associated with delayed bleeding (*P* < 0.20) using univariable analyses were included in the multivariable logistic regression analysis; probability values less than 0.05 were considered significant. Statistical analyses were performed using SPSS, version 21 (IBM Corporation, New York, US), and R, version 4.0.3 (R Foundation for Statistical Computing, Vienna, Austria).

## Results

**Figure 1:**
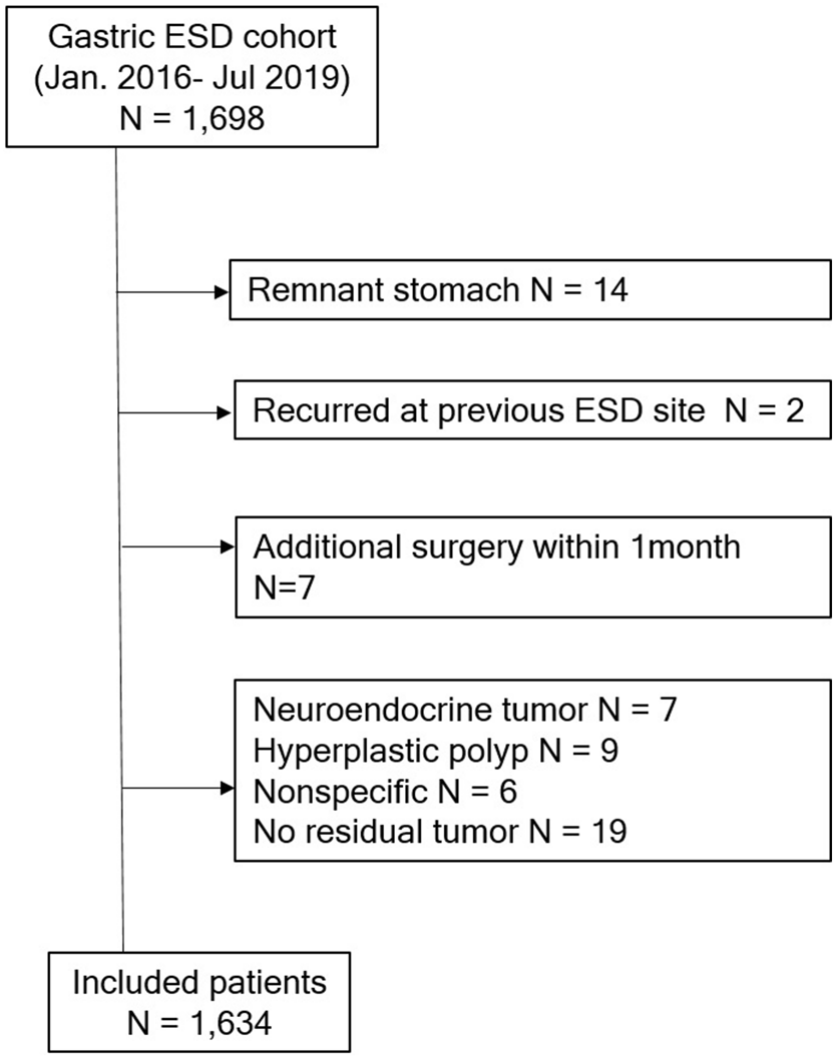
Flow diagram for patient inclusion and exclusion of the study.

A total of 1634 patients were diagnosed with EGC or gastric adenoma, and underwent ESD between January 2015 and July 2019 at SNUH (Fig. 1). The mean patient age was 65.5 years, and a majority of patients were male (1164/1634, 71.2%). Among all cases, 672 (41.1%) were diagnosed with gastric adenoma and 962 (58.9%) were diagnosed with EGC. Forty-seven patients (2.9%) experienced delayed bleeding after ESD. Rates of delayed bleeding according to medication type were 2.1% (32/1499) in the no med group, 8.7% (2/23) in the DOAC group, 14.3% (2/14) in the WFR group, and 11.2% (11/98) in the anti-PLT group. The primary indication for the majority of DOAC users was Afib (22/23, 95.7%); other indications included cerebral infarction (2/23, 8.7%) and pulmonary thromboembolism (1/23, 4.3%). In the DOAC group, 3 patients took 150 mg dabigatran twice per day, 7 patients were treated with 5 mg apixaban twice per day, 10 patients were prescribed 10–20 mg rivaroxaban once daily, and 3 patients were prescribed either 30 mg or 60 mg edoxaban once daily. DOAC users stopped taking their medications 1–8 days (median, 2 days) prior to ESD and resumed their medications 1–5 days (median, 1 day) after ESD. WFR users stopped their medication 2–8 days (median, 4 days) prior to ESD and resumed taking WFR within 4 days (median, 2 days) after ESD. All but two patients underwent a prothrombin time-international normalized ratio (PT-INR) test on the day of admission. The PT-INR was less than 1.5 on the day before ESD.

### Comparison of baseline characteristics between groups

Age was statistically different among the four groups (*P* < 0.001), and patients in the no med group were younger than those in any other patient group (DOAC, *P* < 0.001; WFR, *P* = 0.558; anti-PLT, *P* < 0.001). However, there was no difference with respect to age between the DOAC, WFR, and anti-PLT groups. The percentage of patients with comorbidities (i.e., hypertension, diabetes mellitus, coronary artery disease, chronic heart failure, chronic kidney disease, and stroke) differed among the groups. The DOAC group had a higher rate of chronic heart failure than the no med and anti-PLT groups and a higher rate of stroke than the no med group. Each medication group had higher HAS-BLED scores than the no med group, but there were no differences among the three medication groups. Regarding the laboratory findings of the DOAC group, PT-INR levels revealed an intergroup difference after a post-hoc analysis (no-med, *P* < 0.001; warfarin, *P* = 0.200; anti-PLT, *P* = 0.001) (Table 1). The rate of delayed bleeding after ESD differed among the four groups (*P* < 0.001), and both WFR and anti-PLT users experienced delayed bleeding more than the no med group. The median time of bleeding did not differ between the four groups: DOAC and WFR users experienced bleeding after 4.5 and 6.5 days in median, respectively (range, 1–8 days). However, delayed bleeding occurred median 1 day after ESD in the no med and anti-PLT groups, but occurred between 1–18 days after ESD (Table 2).

**Table 1:** Baseline demographic and clinical characteristics of the patient groups who underwent endoscopic submucosal dissection (ESD) with medication status.

	No med (N = 1499)	DOAC user (N = 23)	Warfarin user (N = 14)	Anti-platelet user (N = 98)	*P*-value
Age (years)	65.04 ± 9.38	71.61 ± 6.11	67.07 ± 9.37	70.30 ± 7.51	< 0.001
**Sex, N (%)**					0.951
Male	1068 (71.2%)	16(69.6%)	11 (78.6%)	69 (70.4%)	
Female	431 (28.8%)	7 (30.4%)	3 (21.4%)	29 (29.6%)	
BMI	24.61 ± 3.08	24.26 ± 2.73	24.88 ± 3.58	25.76 ± 3.21	0.004
**Comorbidity**					
HTN, N (%)	505 (33.7%)	11 (47.8%)	5 (35.7%)	61 (62.2%)	< 0.001
DM, N (%)	272 (18.1%)	5 (21.7%)	6 (42.9%)	36 (36.7%)	< 0.001
DL, N (%)	170 (11.3%)	4 (17.4%)	1 (7.1%)	17 (17.3%)	0.218
CAD, N (%)	63 (4.2%)	4 (17.4%)	4 (28.6%)	27 (27.6%)	< 0.001
CHF, N (%)	2 (0.1%)	3 (13.0%)	3 (21.4%)	0 (0.0%)	< 0.001
CKD, N (%)	33 (2.2%)	1 (4.3%)	3 (21.4%)	2 (2.0%)	0.007
Stroke, N (%)	28 (1.9%)	6 (26.1%)	3 (21.4%)	12 (12.2%)	< 0.001
CLD, N (%)	73 (4.9%)	1 (4.3%)	2 (14.3%)	5 (5.1%)	0.335
A.fib, N (%)	10 (0.7%)	22 (95.7%)	7 (50.0%)	0 (0.0%)	< 0.001
**HAS-BLED score**					< 0.001
< 3	1375 (91.7%)	11 (47.8%)	8 (57.1%)	45 (45.9%)	
≥ 3	124 (8.3%)	12 (52.2%)	6 (42.9%)	53 (54.1%)	
**Location, N (%)**					0.536
Upper third	114 (7.6%)	2 (9.1%)	1 (7.7%)	8 (8.2%)	
Middle third	531 (35.6%)	8 (36.4%)	8 (61.5%)	32 (32.7%)	
Lower third	848 (56.8%)	12 (54.5%)	4 (30.8%)	58 (59.2%)	
Specimen size (cm)	4.15 ± 1.14	4.08 ± 1.35	5.14 ± 2.53	4.32 ± 1.35	0.070
**Invasion depth, N (%)**					0.403
Epithelium	618 (41.2%)	13 (56.5%)	6 (42.9%)	42 (42.9%)	
Lamina propria	385 (25.7%)	5 (21.7%)	2 (14.3%)	23 (23.5%)	
Muscularis mucosa	342 (22.8%)	5 (21.7%)	3 (21.4%)	18 (18.4%)	
Submucosa	154 (10.3%)	0 (0.0%)	3 (21.4%)	15 (15.3%)	
Lymphatic invasion, N (%)	45 (3.0%)	0 (0.0%)	1 (7.1%)	4 (4.1%)	0.479
Vascular invasion, N (%)	8 (0.5%)	0 (0.0%)	0 (0.0%)	1 (1.0%)	0.541
Laboratory finding*					
Hb (g/dL)	13.54 ± 1.48	13.08 ± 1.63	12.64 ± 2.01	13.15 ± 1.63	0.006
PLT (× 10^3^ /μL)	222.33 ± 57.52	192.36 ± 63.18	189.54 ± 58.27	217.57 ± 67.95	0.018
PT (INR)	0.97 ± 0.06	1.07 ± 0.18	1.14 ± 0.22	1.14 ± 0.22	< 0.001
aPTT (sec)	31.17 ± 3.17	32.76 ± 5.19	32.40 ± 2.68	30.72 ± 2.65	0.078

BMI, Body mass index; HTN, Hypertension; DM, Diabetes mellitus; DL, Dyslipidemia; CAD, Coronary artery disease; CHF, Chronic heart failure; CKD, Chronic kidney disease; CLD, Chronic liver disease; A. fib, Atrial fibrillation; ESD, Endoscopic submucosal dissection; Hb, Hemoglobin; PLT, Platelet count; PT, Prothronbin time; INR, International normalized ratio; aPTT, activated partial thromboplastin time.

*Blood tests were done on the day of scheduled admission for ESD procedure. The missing data of Hb, PLT, PT-INR, and aPTT were 81, 92, 136, and 156, respectively.

**Table 2:** Comparison of crude bleeding rate and median time of bleeding between groups divided by medication.

	No med	DOAC	Warfarin	Anti-platelet	*P*-value
Delayed bleeding, N (%)	32/1499 (2.1%)	2/23 (8.7%)	2/14 (14.3%)	11/98 (11.2%)	< 0.001
Time of bleeding, median (range), days	1.0 [0.0–18.0]	4.5 [1.0–8.0]	6.5 [6.0–7.0]	1.0 [0.0–15.0]	0.251
RBC transfusion, N (%)	22 (1.5%)	1 (4.3%)	2 (14.3%)	8 (8.2%)	< 0.001
Second look EGD, N (%)	28 (1.9%)	0 (0.0%)	2 (14.3%)	7 (7.1%)	0.002
Hemostasis, N (%)	14 (0.9%)	0 (0.0%)	1 (7.1%)	3 (3.1%)	0.040
Thromboembolic event, N (%)*	1 (0.1%)	0 (0.0%)	0 (0.0%)	0 (0.0%)	

DOAC, Direct oral anticoagulant; RBC, Red blood cell; EGD, Esophagogastroduodenoscopy.

*Thromboembolic event of acute coronary syndrome occurred 2 months after undergoing ESD which was less likely to be related to the procedure.

### Risk of delayed bleeding after propensity score matching

After propensity score matching, age, sex, and comorbidities did not differ between the DOAC and no med groups. The rates of delayed bleeding were 2/20 (10.0%) in the DOAC group and 1/60 (1.7%) in the no med group (*P* = 0.160). The rates of RBC transfusion, second look endoscopic findings, and endoscopic hemostasis were not statistically different between the DOAC group and no the med group (Table 3).

**Table 3:** Baseline characteristics of direct oral anticoagulant (DOAC) users and patients without any medication.

	Unmatched			Propensity score matching (1:3 Matched)		
	No med (N = 1499)	DOAC user (N = 23)	*P*-value	No med (N = 60)	DOAC user (N = 20)	p-value
Age (years)	65.04 ± 9.38	71.61 ± 6.11	< 0.001	70.6 ± 6.3	71.0 ± 6.0	0.821
**Sex, N (%)**			0.820			0.247
Male	1068 (71.2%)	16 (69.6%)		47 (78.3%)	13 (65.0%)	
Female	431 (28.8%)	7 (30.4%)		13 (21.7%)	7 (35.0%)	
BMI	24.61 ± 3.08	24.26 ± 2.73	0.585	31.1 ± 3.0	33.3 ± 5.2	0.388
**Comorbidities**						
HTN, N (%)	505 (33.7%)	11 (47.8%)	0.183	35 (58.3%)	9 (45.0%)	0.314
DM, N (%)	272(18.1%)	5 (21.7%)	0.592	20 (33.3%)	4 (20.0%)	0.399
DL, N (%)	170 (11.3%)	4 (17.4%)	0.324	12 (20.0%)	3 (15.0%)	0.750
CAD, N (%)	63 (4.2%)	4 (17.4%)	0.016	7 (11.7%)	4 (20.0%)	0.454
CKD, N (%)	33 (2.2%)	1 (4.3%)	0.408	7 (11.7%)	1 (5.0%)	0.672
Stroke, N (%)	28 (1.9%)	6 (26.1%)	< 0.001	5 (8.3%)	5 (25.0%)	0.110
CLD, N (%)	73 (4.9%)	1 (4.3%)	1.000	9 (15.0%)	1 (5.0%)	0.437
**HAS-BLED score**			< 0.001			
< 3	1375 (91.7%)	11 (47.8%)				
≥ 3	124 (8.3%)	12 (52.2%)				
Pathology			0.323			0.783
Adenoma	611 (40.8%)	13 (56.5%)		33 (55.0%)	10 (50.0%)	
AdenoCa W/D, M/D	820 (54.7%)	10 (43.5%)		25 (41.7%)	10 (50.0%)	
AdenoCa P/D	68 (4.5%)	0 (0.0%)		2 (3.3%)	0 (0.0%)	
**Location, N (%)**			0.841			0.865
Upper third	114 (7.6%)	2 (9.1%)		6 (10.0%)	2 (10.0%)	
Middle third	531 (35.6%)	8 (36.4%)		20 (33.3%)	8 (40.0%)	
Lower third	848 (56.8%)	12 (54.5%)		34 (56.7%)	10 (50.0%)	
Specimen size (cm)	4.15 ± 1.14	4.08 ± 1.35	0.755	23.9 ± 2.6	24.5 ± 2.6	0.950
**Invasion depth, N (%)**			0.299			0.907
Epithelium	618 (41.2%)	13 (56.5%)		33 (55.0%)	10 (50.0%)	
Lamina propria	385 (25.7%)	5 (21.7%)		11 (18.3%)	5 (25.0%)	
Muscularis mucosa	342 (22.8%)	5 (21.7%)		14 (23.3%)	5 (25.0%)	
Submucosa	154 (10.3%)	0 (0.0%)		3 (2 (3.3%)	0 (0.0%)	
**Ulcer**						
Present	46 (3.1%)	2 (8.7%)	0.162	3 (5.0%)	1 (5.0%)	1.000
Lymphatic invasion, N (%)	45 (3.0%)	0 (0.0%)	1.000	0 (0.0%)	0 (0.0%)	
Vascular invasion, N (%)	8 (0.5%)	0 (0.0%)	1.000	0 (0.0%)	0 (0.0%)	
**Laboratory finding**						
Hb (g/dL)	13.54 ± 1.48	13.08 ± 1.63	0.145	13.3 ± 1.9	12.8 ± 1.6	0.350
PLT (× 10^3^/μL)	222.33 ± 57.52	193.36 ± 63.18	0.016	206.0 ± 55.9	190 ± 65.4	0.303
PT (INR)	0.9675 ± 0.61	1.07 ± 0.18	0.023	0.97 ± 0.05	1.08 ± 0.19	0.028
aPTT (sec)	31.17 ± 3.17	32.76 ± 5.19	0.214	31.1 ± 3.0	33.3 ± 5.2	0.122
Delayed bleeding, N (%)	32 (2.1%)	2 (8.7%)	0.091	1 (1.7%)	2 (10.0%)	0.160
RBC transfusion, N (%)	22 (1.5%)	1 (4.3%)	0.297	2 (3.3%)	2 (10.0%)	0.259
Second look EGD, N (%)	28 (1.9%)	0 (0.0%)	1.000	3 (5.0%)	0 (0.0%)	0.569
Hemostasis, N (%)	14 (0.9%)	0 (0.0%)	1.000	1 (1.7%)	0 (0.0%)	1.000

BMI, Body mass index; HTN, Hypertension; DM, Diabetes mellitus; DL, Dyslipidemia; CAD, Coronary artery disease; CHF, Chronic heart failure; CKD, Chronic kidney disease; CLD, Chronic liver disease; AdenoCa, Adenocarcinoma; W/D, Well differentiated; M/D, Moderately differentiated; P/D, Poorly diferentiated; Hb, Hemoglobin; PLT, Platelet count; PT, Prothronbin time; INR, International normalized ratio; aPTT, activated partial thromboplastin time; RBC, Red blood cell; EGD, Esophagogastroduodenoscopy.

### Factors associated with delayed bleeding after ESD

We divided patients into two groups according to whether or not they experienced delayed bleeding after ESD. Patients who experienced bleeding had a lower BMI (*P* = 0.002) and a higher prevalence of hypertension, coronary artery disease, chronic heart failure, chronic kidney disease, and chronic liver disease. Patient laboratory data including levels of hemoglobin, platelets, PT-INR, and activated partial thromboplastin time did not differ between the groups, but the percentage of patients who had taken an anticoagulation or anti-PLT agent was higher in the group that experienced delayed bleeding after ESD (*P* < 0.001). A higher percentage of target lesions in the group with delayed bleeding was located in the lower stomach (*P* = 0.025); the size of the specimen was larger in this group as well (*P* = 0.001). (Table 4) We evaluated unadjusted and adjusted odds ratios (ORs) for delayed bleeding by logistic regression analysis to investigate the influence of medication on bleeding. Results of the multivariable prediction model revealed that taking a DOAC was not statistically associated with post-ESD bleeding after adjusting for age, sex, comorbidities, and characteristics of the target lesion (adjusted OR, 2.4; 95% CI, 0.410–13.725; *P* = 0.335) (Table 5). Clinical features of 23 DOAC users were shown below (Supplementary Table 1).

**Table 4:** Comparison of baseline characteristics of patients without bleeding event and patients with delayed bleeding after endoscopic submucosal dissection (ESD).

	No bleeding (N = 1587)	Delayed bleeding (N = 47)	*P*-value
Age (years)	65.42 ± 9.338	66.77 ± 9.796	0.332
**Sex, N (%)**			0.247
Male	1127 (71.4%)	29 (63.0%)	
Female	451 (28.6%)	17 (37.0%)	
BMI	24.71 ± 3.10	23.64 ± 2.92	0.020
**Comorbidities**			
HTN, N (%)	558 (35.2%)	24 (51.1%)	0.030
DM, N (%)	311 (19.6%)	8 (17.0%)	0.852
DL, N (%)	182 (11.5%)	10 (21.3%)	0.061
CAD, N (%)	89 (5.6%)	9 (19.1%)	0.001
CHF, N (%)	6 (0.4%)	2 (4.3%)	0.020
CKD, N (%)	35 (2.2%)	4 (8.5%)	0.024
Stroke, N (%)	45 (2.8%)	4 (8.5%)	0.053
CLD, N (%)	73 (4.6%)	8 (17.0%)	0.002
**Location, N (%)**			0.025
Upper third	125 (7.9%)	0 (0.0%)	
Middle third	566 (35.85)	13 (27.7%)	
Lower third	888 (56.2%)	34 (72.3%)	
Specimen size (cm)	4.15 ± 1.16	4.92 ± 1.46	0.001
**Histology**			0.549
Tubular adenoma	655 (97.5%)	17 (36.2%)	
Tubular/papillary adenocarcinoma	932 (96.9%)	30 (3.1%)	
**Invasion depth, N (%)**			0.866
Epithelium	74 (7.5%)	2 (6.7%)	
Lamina propria	394 (39.9%)	10 (33.3%)	
Muscularis mucosa	354 (35.8%)	12 (40.0%)	
Submucosa	166 (16.8%)	6 (20.0%)	
Lymphatic invasion, N (%)	46 (2.9%)	4 (8.5%)	0.053
Vascular invasion, N (%)	9 (0.6%)	0 (0.0%)	1.000
**Medication, N (%)**			< 0.001
No med	1467 (92.4%)	32 (68.1%)	
DOAC	21 (1.3%)	2 (4.3%)	
Warfarin	12 (0.8%)	2 (4.3%)	
Anti-platelet	87 (5.5%)	11 (23.4%)	
Hb (g/dL)	13.52 ± 1.48	12.92 ± 2.18	0.067
PLT (× 10^3^/μL)	221.69 ± 58.03	210.68 ± 69.91	0.203
PT (INR)	0.98 ± 0.38	0.99 ± 0.08	0.832
aPTT (sec)	31.15 ± 3.12	31.93 ± 4.43	0.242

BMI, Body mass index; HTN, Hypertension; DM, Diabetes mellitus; DL, Dyslipidemia; CAD, Coronary artery disease; CHF, Chronic heart failure; CKD, Chronic kidney disease; CLD, Chronic liver disease; ESD, Endoscopic submucosal dissection; DOAC, Direct oral anticoagulant; Hb, Hemoglobin; PLT, Platelet count; PT, Prothrombin time; INR, International normalized ratio; aPTT, activated partial thromboplastin time.

**Table 5:** The influence of the antiplatelet agent or anticoagulation on delayed bleeding after endoscopic submucosal dissection: univariable and multivariable logistic regression analysis with multivariable odds ratio.

	Univariable			Multivariable (Model 1)^a^			Multivariable (Model 2)^b^		
	OR	95% CI	*P*-value	OR	95% CI	*P*-value	OR	95% CI	P-value
**Medication**									
No med	1 (ref)			1 (ref)			1 (ref)		
DOAC	4.366	0.982–19.413	0.053	3.693	0.796–17.141	0.095	2.371	0.410–13.725	0.335
Warfarin	7.641	1.642–35.547	0.010	4.723	0.858–25.991	0.074	1.418	0.153–13.173	0.759
Anti-PLT	5.796	2.826–11.889	< 0.001	5.083	2.286–25.991	0.074	4.767	2.018–11.260	< 0.001
**Comorbidity**									
HTN	1.924	1.076–3.441	0.027	2.023	1.083–3.805	0.027	1.639	0.826–3.252	0.158
CAD	3.986	1.869–8.502	< 0.001	2.349	1.001–5.512	0.050	3.104	1.26507.616	0.013
CLD	4.254	1.919–9.432	< 0.001	4.087	1.651–10.118	0.002	4.492	1.793–11.258	0.001
Specimen size	1.437	1.215–1.699	< 0.001				1.549	1.282–1.872	< 0.001
**Location**									
Upper-middle	1 (ref)						1 (ref)		
Lower	2.035	1.066–3.886	0.031				2.402	1.195–4.826	0.014

OR, Odds ratio; CI, Confidence interval; DOAC, Direct oral anticoagulant; Anti-PLT, Anti-platelet; HTN, Hypertension; CAD, Coronary artery disease; DL, Dyslipidemia; CHF, Chronic heart failure; CKD, Chronic kidney disease; CLD, Chronic liver disease.

^a^BMI, Comorbidities (HTN, DL, CAD, CHF, CKD, CLD, Stroke, Malignancy), medication were adjusted in model 1.

^b^Model 1 + specimen size, location, lymphatic invasion were adjusted in model 2.

## Discussion

This large-scale, single tertiary center, retrospective study was designed to investigate the influence of DOACs on delayed bleeding after gastric ESD. Although there are several retrospective studies that evaluated delayed bleeding rate after gastric ESD in DOAC users, evidences still lacks to support the current guideline^18–22^. Due to retrospective design, basic characteristics of the study population and periprocedural drug management was different among previous studies.

Although the results were not statistically significant, the rates of delayed bleeding in DOAC users were higher than those of the no med group (8.7% vs 2.1%, *P* = 0.091). This tendency remained the same in the 1:3 propensity score-matched subcohort (10.0% vs 1.7%, *P* = 0.160). After multivariable logistic regression analysis, the use of DOAC increased the risk of delayed bleeding after ESD 2.4 fold compared to the no med group, although these results were not statistically significant. DOAC use appears to confer a minor increased risk of delayed bleeding compared to the no med group. This increased risk of post-ESD bleeding could be influenced by an underlying condition in addition to DOAC use.

After consideration of both half-life of DOAC and the patient’s creatinine clearance, DOACs are generally discontinued at least 1–3 days for procedures associated with a high risk of bleeding^8–10^. DOAC users in this study stopped taking their medications 2 days (median) prior to ESD. Delayed bleeding after ESD occurred in 8.7% (2/23) of DOAC users; those patients had normal renal function and complied with the recommended guidelines for DOAC cessation. The appropriate timing of reinitiation of anticoagulation is essential to balance the risks of thrombosis and bleeding. There are no data about the optimal timing for the reinitiation of DOACs after ESD. DOAC users in this study resumed their medications 1 day (median) after ESD. In two cases of post-ESD bleeding among DOAC users, one case occurred when the patient had skipped seven doses of rivaroxaban without restarting this medication, and the other case occurred 1 week after the reinitiation of apixaban. In both cases, the time of drug reinitiation was 1 week after ESD. Additional cases of post-ESD bleeding in DOAC users should be collected to correlate the reinitiation of DOAC use with bleeding risk. In this study, some anti-PLT users experienced delayed bleeding up to 2 weeks after the reinitiation of the anti-PLT agent, but for patients administered an anticoagulation therapy, bleeding occurred within 1 week after reinitiation. On the day of hospital discharge, users of anti-thrombotic agents should be told that they have a prolonged risk for bleeding compared to those not taking medications.

There were no thromboembolic events related to DOAC cessation in DOAC users with Afib (22/23) despite high CHA_2_DS_2_-Vasc scores (mean, 3.4; standard deviation, 1.4). The reported risk of stroke in Afib patients who have undergone endoscopy after cessation of warfarin (prescribed for primary stroke prevention) is 1%^23^. There was one case of a thromboembolic event in the no med group 2 months after ESD, but no cases of thromboembolic events in groups taking any anti-thrombotic agents. A previous study showed that 7% (14/197) of cardioembolic infarctions occurred after the discontinuation of WFR prior to the medical procedures^24^. Additional data are required to assess the risk of cardioembolic events in patients who stop taking DOACs prior to endoscopic procedure.

The multivariable analysis of the current study revealed that the comorbidities of coronary artery disease and chronic liver disease were associated with delayed bleeding after ESD. Anti-PLT agents are essential for secondary prophylaxis in patients with coronary artery disease; these agents are restarted soon after ESD, and a higher percentage of these patients require dual anti-PLT therapies compared to those without coronary artery disease. This observation may explain why coronary artery disease is associated with delayed bleeding after adjustments for medication use. Among the 81 patients who had chronic liver disease, 39 cases were liver cirrhosis (LC) from any cause, and the other 42 cases were non-cirrhotic liver disease (18 Fatty liver disease, 22 chronic hepatitis B, and 2 chronic hepatitis C). LC patients had lower platelet counts and a higher INR. Although all patients with cirrhosis in this study were in a compensated state, these baseline laboratory values differed from those patients without LC, which could have influenced the risk of post-ESD bleeding. Also, impaired platelet function and rebalanced fibrinolysis could lead to impaired hemostasis and increased post-ESD bleeding^25–27^. Clinical features of the target lesion for ESD, including a larger specimen size, the presence of an ulcer, and a lower location, were suggested factors associated with post-ESD bleeding in previous studies^28–34^. In this study, tumor size estimated by endoscopic finding was unrelated to post-ESD bleeding, but specimen size, which is highly correlated with the size of iatrogenic ulcers after ESD, was associated with delayed bleeding.

This study has some limitation. First, the number of DOAC users who underwent ESD was relatively small. However, the prevalence of Afib in this study was 0.99% (4/403) in patients younger than 60 years of age, 2.02% (13/643) in those between 60 and 69 years of age, and 3.74% (22/588) in patients aged 70 years and older. Considering the overall prevalence of Afib in Korea and the increasing prevalence of Afib with age according to previous reports, the sample size of this study might accurately represent the Korean population^35,36^. Furthermore, the mean CHA_2_DS_2_-Vasc score of patients with Afib in this study was 3.07, which represents the same thrombotic risk as that found in a nationwide study^37^. Second, this study is retrospective in design and was performed at a single tertiary center, which might have resulted in selection bias. However, the similar prevalence of Afib and similar CHA_2_DS_2_-Vasc scores as observed in previous studies suggests that the baseline characteristics of this study reflect those of the general population. Considering the rapidly aging of society, the prevalence of Afib and the use of DOAC are estimated to increase as well. Lastly, due to the small number of users of anti-thrombotic agents, the only one case of thromboembolism occurred in no med group. However, considering the CHA_2_DS_2_-Vasc scores (mean, 3.4; standard deviation, 1.4) of DOAC users with Afib and the adjusted stroke rate, we cannot conclusively say that this single thrombotic event is significant^38^. Additional data are needed to answer this question.

In conclusion, the rate of delayed bleeding in DOAC users was higher than that of the no med group and this remained the same after propensity score matching. In multivariable analyses adjusted for age, sex, comorbidities, and tumor characteristics, the differences in the rate of delayed bleeding between no med and DOAC groups became smaller, which suggests that the slight differences observed were the result of underlying patient conditions in addition to medication use. Additional data are needed to determine when to stop and resume DOACs during endoscopic procedures have a higher risk of bleeding without increasing thromboembolic risk.

## Supplementary Information

Supplementary Information
