# Supplementary material for: Use of direct oral anticoagulants does not significantly increase delayed bleeding after endoscopic submucosal dissection for early gastric neoplasms
Source: Sci Rep. 2021 Apr 30;11:9399. doi: 10.1038/s41598-021-88656-z (PMC8087783; doi:10.1038/s41598-021-88656-z)
Supplement: Supplementary file 1 — Supplementary Information [file 41598_2021_88656_MOESM1_ESM.pdf]

Title: Use of direct oral anticoagulants does not significantly increase delayed bleeding after endoscopic submucosal dissection for early gastric neoplasms

Authors

Jinju Choi M.D.[1], Soo-Jeong Cho M.D. PhD*[1], Sang-Hoon Na M.D. PhD[2], Ayoung Lee M.D.[1], Jue Lie Kim M.D.[1], Hyunsoo Chung M.D. PhD[1], Sang Gyun Kim M.D. PhD[1]

Supplementary table 1. Clinical features of all patients who had been taken direct oral anticoagulant (DOAC) before and after endoscopic submucosal dissection (ESD)

Case No.	Sex/Age	Co-morbidities	DOAC	Date of D/C (days before ESD)	Date of restart (days after ESD)	Histology	Location	Specimen size, (cm)	Time of bleeding after ESD	pRBC transfusion, units	Second look EGD	Endoscopic hemostasis	Additional hospital stay (day)
1	F/70	HTN, DM, Stroke, Afib	Rivaroxaban 15mg qd	4	4	AdenoCa WD	Lower	5.5	1	4	-	-	3
2	F/73	HTN, Afib	Apixaban 5mg bid	1	1	AdenoCa MD	Lower	5.0	8	2	-	-	1
3	M/73	CAD, Stroke, Prostate cancer, Afib	Rivaroxaban 15mg qd	8	2	TAHG	Middle	4.0					
4	F/74	Afib	Rivaroxaban 15mg qd	2	1	TAHG	Middle	3.2					
5	F/65	HTN, DL, CAD, Afib	Apixaban 5mg bid	3	2	TALG	Lower	4.7					
6	M/65	HTN, Afib	Dabigatran 150mg bid	2	2	TALG	Middle Middle	4.0					
7	M/75	CHF, Afib	Apixaban 5mg bid	1	1	AdenoCa MD		3.0					
8	M/76	PTE, Lung cancer	Rivaroxaban 10mg qd	3	2	AdenoCa WD	Upper	3.8					
9	M/69	HTN, DM, Afib, CKD, stroke, HCC	Dabigatran 150mg bid	2	1	TALG	Lower	4.4					
10	F/77	HTN, DL, Afib	Edoxaban 30mg qd	1	1	AdenoCa WD	Lower	4.2					
11	M/68	HTN, DM, DL, Afib	Rivaroxaban 20mg qd	2	1	TALG	Lower	3.5					
12	M/77	Afib	Apixaban 5mg bid	2	1	TALG	Middle	3.0					
13	M/74	Stroke, Afib, HCC	Rivaroxaban 20mg qd	4	1	AdenoCa WD	Lower	4.0					
14	M/74	HTN, DL, CHF, Afib	Apixaban 5mg bid	2	2	AdenoCa MD	Lower	4.7					
15	M/80	Stroke	Dabigatran 150mg bid	2	unknown	TALG	Lower	3.0					
16	F/68	CAD, Afib	Apixaban 5mg bid	2	1	TALG	Middle	3.4					
17	M/69	HTN, DM, Afib	Rivaroxaban 20mg qd	1	1	AdenoCa WD	Middle	4.9					

DOAC, Direct oral anticoagulant; D/C, Discontinuation; ESD, Endoscopic submucosal dissection; pRBC, pack RBC; EGD, Esophagogastroduodenoscopy; HTN, Hypertension; DM, Diabetes mellitus; DL,

Dyslipidemia; CAD, Coronary artery disease; CHF, Chronic heart failure; CKD, Chronic kidney disease; CLD, Chronic liver disease; Afib, Atrial fibrillation; PTE, Pulmonary thromboembolism; HCC, Hepatocellular carcinoma; ESD, Endoscopic submucosal dissection; DOAC, Direct oral anticoagulant; qd, quaque die; bid, bis in die; TALG, tubular adenoma low brade; TAHG, tubular adenoma high grade; adenoCa, adenocarcinoma; WD, well differentiated; MD, moderately differentiated; PD, poorly differentiated;
